# Progress in the clinical detection of heterogeneity in breast cancer

**DOI:** 10.1002/cam4.943

**Published:** 2016-10-24

**Authors:** Jun‐Long Song, Chuang Chen, Jing‐Ping Yuan, Sheng‐Rong Sun

**Affiliations:** ^1^ Department of Breast and Thyroid SurgeryRenmin Hospital of Wuhan UniversityWuhanHubei430060China; ^2^ Department of PathologyRenmin Hospital of Wuhan UniversityWuhanHubei430060China

**Keywords:** Breast cancer, genomic analysis, heterogeneity, imaging analysis, pathologic analysis

## Abstract

Breast cancer is currently the most common form of cancer and the second‐leading cause of death from cancer in women. Though considerable progress has been made in the treatment of breast cancer, the heterogeneity of tumors (both inter‐ and intratumor) remains a considerable diagnostic and prognostic challenge. From clinical observation to genetic mutations, the history of understanding the heterogeneity of breast cancer is lengthy and detailed. Effectively detecting heterogeneity in breast cancer is important during treatment. Various methods of depicting this heterogeneity are now available and include genetic, pathologic, and imaging analysis. These methods allow characterization of the heterogeneity of breast cancer on a genetic level, providing greater insight during the process of establishing an effective therapeutic plan. This study reviews how the understanding of tumor heterogeneity in breast cancer evolved, and further summarizes recent advances in the detection and monitoring of this heterogeneity in patients with breast cancer.

## Introduction

Breast cancer, an ancient disease first noted by the Egyptians more than 3500 years ago 1, is the most common cancer and the second‐leading cause of death from cancer in women in the United States 2. About 12% of women in the United States will be diagnosed during their lives with breast cancer, and it is estimated that more than 40,000 patients will die from breast cancer in 2016 in the United States 2. The probability of receiving a breast cancer diagnosis is only 1.9% in women under the age of 50 years 2; most women receive such a diagnosis after this age, accounting for nearly 80% of all cases of breast cancer 3. Though considerable progress has occurred in the treatment of breast cancer, many treatment challenges remain. One of these challenges is overcoming the clinical heterogeneity of the disease. Breast cancer was initially thought to be homogeneous; clinical observation eventually revealed otherwise, leading to the prognosis varying from patient to patient. Cooper observed that the size of breast tumors changed with the menstrual cycle 4. Beatson further found that removal of the ovaries could reduce the tumor size of breast cancer 5. Currently, considerable effort is being devoted to exploring and defining the mechanisms underlying the clinical heterogeneity of breast cancer. Steinthal employed clinical staging to identify this clinical heterogeneity 6. The development of pathologic techniques has allowed physicians and patients to obtain more information about the heterogeneity of the tumors associated with breast cancer on the cellular and tissue level. For example, Greenough applied histologic classification to assess the differences in tumor differentiation and proliferation among patients 7. Understanding of the heterogeneity of breast cancer has been deepened by the identification of different expression levels of the estrogen receptor (ER), the progesterone receptor (PR), and human epidermal growth factor receptor‐2 (Her‐2), each of which is a key biomarker used for clinical decision making.

During the past two decades, the rapid development of genetic analysis technologies has enabled depiction of the heterogeneity of breast cancer on a genetic level 8. Several genetic alterations have been identified, including germline BRCA mutations, which frequently occur in hereditary breast cancer 9. Based on comprehensive gene‐expression profiling, breast cancer has been classified into five main categories: luminal A, luminal B, Her2‐enriched (also called Her2‐related), claudin‐low, and basal‐like 10.

**Figure 1:**
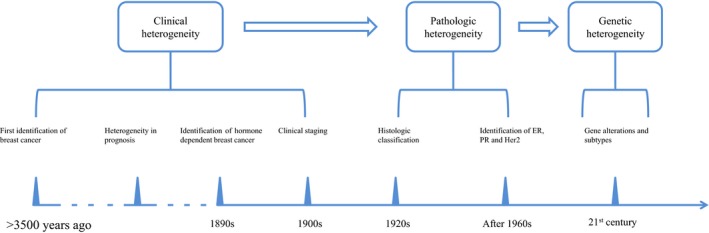
Detailed history of the understanding of the heterogeneity of breast cancer. At first, breast cancer heterogeneity could only be assessed according to its clinical characteristics (stage, recurrence, metastasis). Technological advances provided further information on the pathologic heterogeneity in histologic classification and the expression of key molecules. During the past two decades, the rapid development of genetic analysis has enabled depiction of the heterogeneity of breast cancer on a genetic level.

From clinical observation to testing for genetic mutations, a lengthy and steep learning curve has been experienced in understanding the tumor heterogeneity of breast cancer (Fig. 1). Various studies have demonstrated that heterogeneity can occur either among different patients with the same tumor type (intertumor heterogeneity) or within the same patient (intratumor heterogeneity) 11, 12, 13. Traditionally, intertumor heterogeneity was thought to be the largest barrier in the treatment of breast cancer. Effective and individualized therapeutic plans have been established based on the understanding of intertumor heterogeneity. For example, adjuvant endocrine therapy is widely prescribed for patients who have hormone receptor‐positive tumors. However, apart from intertumor heterogeneity, it should be noted that intratumor heterogeneity also poses a tremendous challenge for treatment selection. In fact, ER, PR, and Her‐2 are expressed differently in different regions within the same tumor as well as between the matched primary tumors and metastatic lesions 14. To further add to this complexity, microenvironmental components of tumors (such as stromal cells and extracellular matrix) are highly variable among different patients 15 and impact the effectiveness of treatment. In the clinical setting, patients' tumors may have the same molecular classification and receive the same treatment, yet patients may experience very different outcomes. Some patients acquire resistance to therapies such as endocrine therapy and targeted therapy during treatment in spite of the initial efficacy of these approaches 16, 17. Therefore, it is important to identify potential intertumor heterogeneity and intratumor heterogeneity to devise new treatment plans with new therapeutic targets.

**Figure 2:**
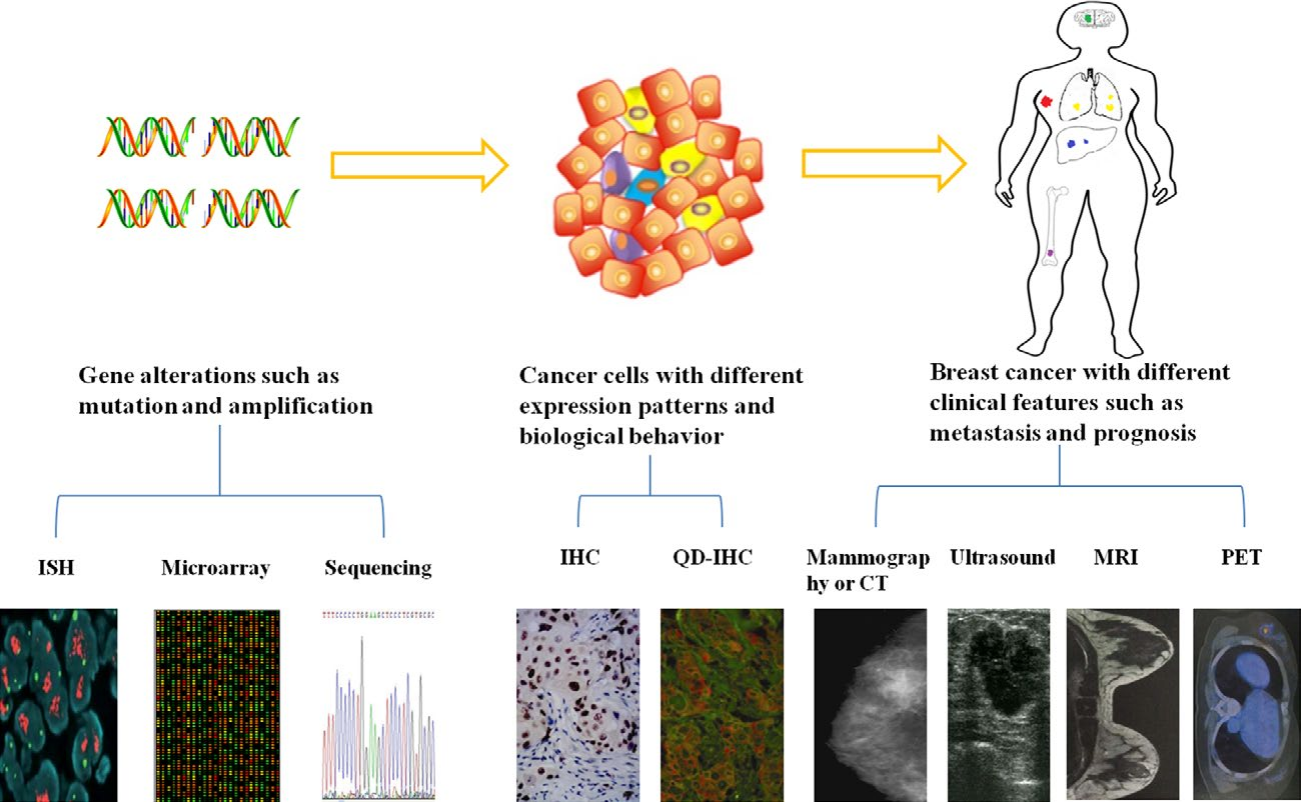
Heterogeneity can be detected on three levels: genes, cells and tissues, and clinical features. Genetic heterogeneity can be detected using gene sequencing, microarrays, and ISH. Pathologic heterogeneity can be detected with the help of IHC and QDs‐IHC. Heterogeneity can be determined through the clinical features of tumors using various imaging methods such as mammography, ultrasound, MRI, and PET. ISH: In situ hybridization; IHC: immunohistochemistry; QDs: quantum dots; CT: computed tomography; MRI: magnetic resonance imaging; PET: positron‐emission tomography. All of the pictures were produced at our clinic's imaging center.

In previous studies, cumulative exposure to carcinogenic factors led to elevations in chromosomal instability and cancer‐specific driver mutations 18, 19, both of which lead to abnormal gene expression 12, 20, 21. The genetic alterations might be different across cancer cell subgroups. Each of these alterations (alone or in different combinations) endows different cancer cell subgroups with different features 22, 23; cancer cell subgroups form solid tumors with distinct clinical (i.e., morphologic and prognostic) features. Therefore, using the origins of heterogeneity as a starting point, both intertumor and intratumor heterogeneity can be detected. Powerful technologies such as genomic analysis technology, molecular and pathologic technology, and imaging techniques have been used to detect breast cancer heterogeneity and inspect its dynamic changes (Fig. 2). This review summarizes recent advances in detecting and monitoring heterogeneity in breast cancer and is split into three sections, each of which reviews one of these technologies. At the end of this review, we will discuss how these new techniques have improved our understanding of the heterogeneity of breast cancer and its clinical management.

## Genomic Analysis

Genomic analysis is an important tool for analyzing the heterogeneity of tumors. As early as 1978, karyotyping was used for the detection of genomic abnormalities 24. However, because DNA samples derived from a breast tumor are a mixture of different DNA from heterogeneous tumor cells, it was difficult to identify the degree of genomic heterogeneity of cancer cells. Considerable progress has occurred with respect to DNA sequencing and the development of detection methods for genomic abnormalities. Recent technological advances have included the microarray, next‐generation sequencing (NGS), and in situ sequencing. Each of these captures the genomic diversity of breast cancer cells.

## In situ Hybridization

In situ hybridization (ISH), a common technique widely used to detect gene number copy alterations, localizes a specific DNA or RNA sequence in tissue by using labeled probes with known sequences 25. ISH plays an important role in detecting both intertumor and intratumor gene heterogeneity in breast cancer. For example, the fluorescence in situ hybridization (FISH) assay is considered the “gold standard” in evaluating HER2 gene status in patients with breast cancer 26. The FISH assay has also been widely used to identify gene copy number and distribution heterogeneity. For example, Janiszewska and colleagues employed FISH to assess the spatial heterogeneity of cellular genetic diversity and the changes in the frequency and topology of the PIK3CA mutation and Her‐2 amplification within Her‐2‐positive breast cancer during neoadjuvant therapy 27. Furthermore, the development of the multicolor FISH (mFISH) assay makes it possible to simultaneously detect the degree of intertumor and intratumor heterogeneity in several genes 28, 29. Li and colleagues used mFISH to detect copy number aberrations in four genes related to cell cycles in patients with breast cancer 29. More recently, ISH was employed to identify heterogeneous expression in RNA molecules in breast cancer, providing information about tissue‐specific and cell‐specific expression 30.

## Gene‐Expression Profiling

Gene‐expression profiling is an important method of detecting gene‐expression heterogeneity in patients with breast cancer. Genetic microarrays and 21‐gene expression assays are the most frequently used techniques in clinical detection of RNA‐expression heterogeneity among patients.

A *gene microarray* is a collection of microscopic probes attached to a solid surface. The concept was first introduced by Schena in 1995 31, and is based on the ability of DNA to find and spontaneously bind its complementary sequence in a reversible way with high specificity 32. The technique can simultaneously measure the expression levels of thousands of genes and identify differentially expressed genes among different patients with cancer 33, 34. Gene microarrays have been used to identify a number of differentially expressed genes 33. As early as 2000, Perou and colleagues performed pattern analysis for gene expression in breast cancer using complementary DNA microarrays, initially discovering five major intrinsic gene signatures: luminal A, luminal B, Her‐2‐enriched, claudin‐low, and basal‐like 10. Gene microarrays could further be used to predict response to chemotherapy and risk of recurrence by inspecting the expression level of genes related to therapy resistance and recurrence 34, 35. Recently, microarrays have been frequently used to screen noncoding RNAs related to breast cancer and identify new tumor subtype markers 36, 37, 38. Though the microarray can attain the level of high‐throughput analysis of gene expression, it cannot provide in situ information about gene expression.

The 21‐gene expression assay is based on reverse transcriptase polymerase chain reaction (RT‐PCR), which is able to qualitatively detect gene expression. In 2004, Paik and colleagues first developed a scoring system based on 21 prospectively selected genes in paraffin‐embedded tumor tissue to quantify the likelihood of distant recurrence in patients with node‐negative, ER‐positive breast cancer 39. They further demonstrated that the 21‐gene recurrence score could also be a powerful tool to predict the magnitude of benefit from chemotherapy 40. Several additional studies have shown that the use of the 21‐gene expression assay is cost‐effective in patients whose cancer has not metastasized to the lymph nodes 41, 42, 43. The 21‐gene expression assay has been widely used to predict tumor heterogeneity in disease recurrence and response to chemotherapy 44, 45, 46, 47. Various studies have demonstrated that 21‐gene recurrence score changed the clinical–pathological adjuvant chemotherapy recommendation 48, 49, 50, 51, 52, 53.

## Gene Sequencing

In 1977, Frederick Sanger first introduced his DNA sequencing technique 54. After 30 years of development, novel sequencing technologies with reduced costs and increasing throughput have been developed. NGS is a newly developed high‐throughput technology that has revolutionized cancer genome sequencing by providing detailed characterization of the cancer genome and epigenomic information about breast cancer 55, 56, 57. NGS makes it possible to perform large‐scale analysis of cancer genes, leading to the discovery of new genes associated with breast cancer and of the heterogeneity of individual tumors 58. Stephens and colleagues identified several new cancer genes in breast cancer with the help of NGS 18. Furthermore, based on the NGS results, additional bioinformatic tools will allow mining of the sequencing data and unveiling of the evolution process for cancer cell subgroups within breast cancer 59. The phylogenetic tree for breast cancer evolution provides an intuition into the dynamic evolutionary course of intratumor heterogeneity 22, 23, 60. In combination with flow‐based cell sorting and efficient whole‐genome amplification (WGA), NGS can be used to detect the genomic alteration of single tumor cells 22. NGS‐based liquid biopsy is currently an area of great research interest and will ideally facilitate the early diagnosis of intratumor heterogeneity and continuing surveillance of dynamic changes 61, 62, 63. Murtaza and colleagues successfully inspected the dynamic changes in genomic architecture derived from circulating tumor cells during adjuvant chemotherapy and identified genes related to resistance to chemotherapy 64. Although NGS can roughly identify spatial heterogeneity in genomic alterations through multiregional sequencing, it cannot provide information about in situ gene expression 65, 66.

Recently, Lee et al. developed a new technique, fluorescent in situ sequencing of RNA, for gene expression profiling 67, 68 through conversion of RNA into cross‐linked cDNA amplicons and manual sequencing on a confocal microscope. This technique is still in its experimental phase, but has broad prospects for helping researchers further understand intratumor heterogeneity.

## Pathologic Analysis

Pathologic analysis plays important roles in the diagnosis and subtyping of breast cancer. Developments in pathologic technologies have promoted greater understanding of both intertumor and intratumor heterogeneity. Pathologic analysis can now combine morphology with molecular biology, providing new insights into breast cancer heterogeneity.

Pathologic analysis techniques such as immunohistochemistry (IHC) and immunofluorescence (IF) have been widely used for assessing therapeutic biomarkers of breast cancer and identifying subsets of patients with different outcomes 69, 70. IHC markers such as ER, PR, Her‐2, and proliferation markers could divide breast cancer cases into groups remarkably similar to subtypes defined by gene expression studies 71, 72, 73; however, these are qualitative analyses. Quantifying tumor heterogeneity has become more urgent with these developments in pathology's capabilities. Some studies have attempted to conduct semiquantitative analysis using conventional IHC and successfully identified different degrees of intratumor heterogeneity 74, 75. Potts and colleagues combined semiquantitative analysis with ecology diversity statistics to evaluate the heterogeneity on IHC‐stained breast cancer samples and identified new features in HER2 expression among different patients 74.

Though conventional pathologic analysis methods have great value in evaluating the heterogeneity of breast cancer, due to its limitations in quantitative analysis and multimolecular staining, these methods are not able to completely reveal the heterogeneity of breast cancer, especially intratumor heterogeneity.

**Figure 3:**
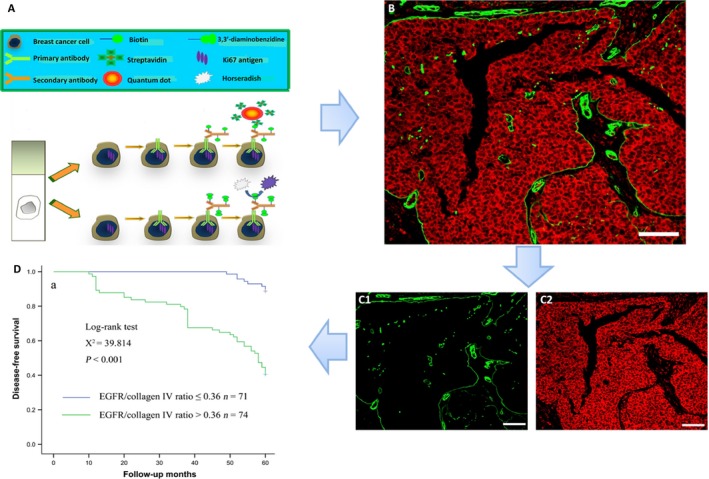
QDs‐based imaging for the study of breast cancer's heterogeneity. Schematic plots of QDs‐based biomarker imaging (A); QDs‐based imaging of multiple biomarkers simultaneously (B: collagen IV [green] and epidermal growth factor receptor [red]); multispectral analysis software allows unmixing and spectral analysis of collagen IV (C) and EGFR (D); new prognostic features could be illustrated by the ratio between collagen IV and EGFR (E). EGFR: epidermal growth factor receptor; QDs: quantum dots. Reproduced with permission from 81, 82.

Recently, nanotechnology has been regarded as a promising tool to illustrate the heterogeneity of breast cancer. Optical‐based nanoparticle imaging, such as quantum dots‐based immunohistochemistry (QDs‐IHC), is an important branch of nanotechnology as applied in medicine. Most of the QDs are semiconductor nanocrystals that have properties including high fluorescence intensity, strong resistance to photobleaching and chemical degradation, size‐tunable emission wavelengths, and simultaneous multiple fluorescence under a single excitation source 76, 77. Due to these properties, QDs‐IHC is a powerful tool to detect breast cancer heterogeneity, as it can provide in situ information for multiple biomarkers (Fig. 3) 78. Chen and colleagues successfully visualized Her‐2 and ER simultaneously with QD‐based imaging; this technique very clearly displayed the heterogeneous expression of these markers in breast cancer 79. Furthermore, Peng and colleagues obtained multidimensional information both from cancer cells and from the tumor microenvironment through QDs‐IHC 80. When performing multiplex imaging of various key immunomarkers such as epidermal growth factor receptor (EGFR), Her‐2, and the Ki‐67 protein simultaneously, we found new tumor characteristics that were associated with breast cancer prognosis 81, 82, 83. Furthermore, Chen and colleagues divided patients with breast cancer into five new subtypes through QD‐based quantitative determination of HER2 and EGFR in combination with hormone receptor status 83. To summarize, QDs‐IHC provides new insight into the heterogeneity of breast cancer and will play an important role in individualized breast cancer treatment.

## Imaging Analysis

**Figure 4:**
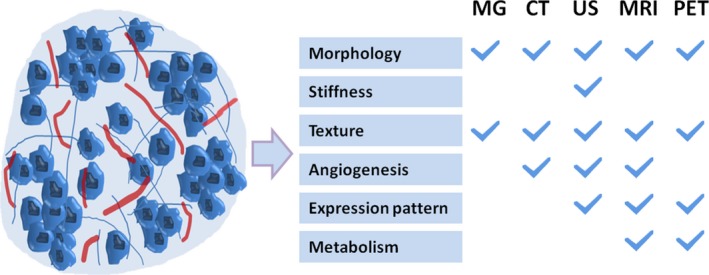
Clinical features demonstrated by different imaging techniques. Clinical heterogeneity can be assessed using various clinical features such as morphology, stiffness, texture, angiogenesis, molecular expression, and metabolism. Different imaging techniques can be used to detect these features. MG: mammography; CT: computed tomography; US: ultrasonography; MRI: magnetic resonance imaging; PET: positron‐emission tomography.

A variety of imaging techniques exist for the screening and diagnosis of breast cancer. Mammography, ultrasound, magnetic resonance imaging (MRI), and positron emission tomography (PET) are the most common techniques for breast cancer imaging. These imaging techniques can reveal the diverse characteristics of breast cancer (Fig. 4). With the development of technology and deeper recognition of breast cancer, some imaging techniques are further used to detect breast cancer heterogeneity. This section summarizes recent advances in this area.

## X‐ray‐Based Imaging

X‐ray‐Based imaging includes techniques such as mammography, computed tomography, and new imaging techniques such as digital breast tomosynthesis (DBT). Mammography has been widely used in breast cancer screening and allows physicians to obtain information about calcifications and breast cancer's morphologic features. The mass shape (round, oval, lobular irregular, and obscured) on mammography has been associated with the Oncotype Dx (a breast cancer array incorporating the mRNA expression of 21 genes) recurrence score 84. However, very little research to date has evaluated the application of mammography in detecting inter‐ or intratumor heterogeneity due to the limited information yielded by the imaging technique.

DBT, first introduced in 2011, improved on standard mammography by increasing breast cancer detection rates 85. However, limited evidence exists of its use in detecting heterogeneity in breast cancer. Computed tomography (CT) allows scanning of the entire breast in thin slices 86, 87. The use of contrast material with CT allows more information to be obtained about the characteristics of breast cancer in a particular patient. Tamaki and colleagues found that CT findings could serve as predictive prognostic factors when correlated with histological characteristics 88. However, due to its radioactivity and low sensitivity, CT is not as commonly used as MRI for detection and staging of breast cancer or for potential determination of heterogeneity.

## Ultrasound

With the development of ultrasound technology, two relatively new techniques, contrast‐enhanced ultrasound (CEUS) and elastography, have added complementary information to breast cancer diagnosis. Aside from morphological features, CEUS can provide more information about tumor angiogenesis.

Elastography is based on the depiction of tissue stiffness, which in breast cancer has been associated with interactions among tumor cells, stromal cells, and the extracellular matrix 89. These properties are associated with heterogeneity in breast cancer, and many authors have tried to use ultrasound to detect breast cancer heterogeneity. Elastography also demonstrated that some sonographic features are associated with pathologic features, including histologic grade 90; however, elastography was not useful for the correlation of mechanical elasticity with breast cancer subtype 90, 91, 92.

CEUS was reported to be a useful tool for correlating imaging features with pathological characteristics. Several studies identified heterogeneity in the expression of vascular endothelial growth factor (VEGF) using CEUS 93, 94. Masumoto and colleagues found that perfusion parameters on CEUS were significantly associated with ER, Her‐2, and Ki‐67 status 95. This result is consistent with other studies 96, 97. CEUS also achieved high accuracy in predicting the heterogeneity of the response of breast cancer to neoadjuvant chemotherapy 98, 99. The use of microbubbles labeled by specific antibodies also allows CEUS to achieve the level of detail of molecular imaging. Sorace and colleagues successfully depicted microvessel density within tumors using multitargeted microbubbles conjugated with antibodies against mouse Av*β*3 integrin, P‐selectin, and vascular endothelial growth factor receptor 2 (VEGFR2) 100. Based on these results, ultrasound will be a powerful tool for detecting both intertumor and intratumor heterogeneity of breast cancer.

## Magnetic Resonance Imaging

Due to its excellent soft‐tissue contrast and high sensitivity, MRI has been widely used in diagnosis of breast cancer as a complementary imaging technique to mammography and ultrasound 101. Parameters such as tumor size, morphology, and shape can be obtained from MR images. Dynamic contrast‐enhanced MRI (DCE‐MRI) even allows the investigation of microvascular structure and kinetic characterization (e.g., maximum relative enhancement, time to peak, area under the curve) as well as statistical measurement of texture enhancement (e.g., gray level co‐occurrence matrix) 102. These parameters reflect intratumor characteristics including growth patterns, angiogenesis, and permeability. Therefore, MRI is a feasible tool for evaluating breast cancer heterogeneity.

Numerous studies have been conducted to explore the application of MRI in screening for breast cancer heterogeneity. Yun and colleagues demonstrated that MRI parameters such as standard deviation and kurtosis of apparent diffusion coefficient (ADC) values were closely related to intratumor spatial heterogeneity of necrosis patterns and vascularity in MCF‐7 and MDA‐MB‐231 xenograft models 103. Different breast cancer subtypes exhibit different growth patterns and angiogenesis 104, 105. Therefore, it is possible to correlate MRI parameters with molecular subtypes of breast cancer. In a retrospective analysis of 102 patients, Chang and colleagues used receiver operating characteristic (ROC) curve analysis in conjunction with MRI to identify region‐based features of tumors and achieve high accuracy in breast cancer classification 106. ADC was also reported to be associated with breast cancer subtypes 107, 108, 109, 110. Some studies further attempted to correlate imaging features with expression information. Sutton and colleagues found that MR‐derived image features (such as kurtosis) were significantly related to the Oncotype Dx recurrence score 111. Other studies have reached similar conclusions 84. MRI is also a powerful tool to predict the heterogeneity of the response to anticancer therapy among patients 112, 113. Ashraf and colleagues determined that DCE‐MRI kinetic statistics could predict response to neoadjuvant chemotherapy 112. Another study explored the use of chemical exchange saturation transfer MRI (CEST‐MRI) to characterize the metabolic heterogeneity within tumors 114. CEST contrast was linearly correlated with nicotinamide adenine dinucleotide hydrate (NADH) concentration and the NADH redox ratio 100. These results might confirm the usefulness of a novel noninvasive imaging surrogate to screen for metabolic heterogeneity in breast cancer.

## Molecular Imaging

Molecular imaging is an emerging discipline with great promise in detecting intertumor and intratumor expression patterns of key molecules in breast cancer. Molecular imaging depends greatly on labeled biomarkers; these can be molecules, proteins, or antibodies. By tracing these labeled biomarkers, we can evaluate heterogeneity in biochemical changes, cellular physiology, cellular function, and metabolism within breast cancer 115.

PET is the most frequently used molecular imaging technique. PET can be used to assess various properties of tumors with an appropriate radiotracer, such as ^18^F‐fluorodeoxyglucose (^18^F‐FDG) for metabolism, ^18^F‐fluorothymidine (^18^F‐FLT) for proliferation, and 18F‐fluoroestradiol (^18^F‐FES) for ER status 115, 116. Therefore, it can be a powerful tool to identify and inspect the dynamic changes in intratumor heterogeneity of breast cancer.

Several studies found that different 18F‐FDG PET features could be translated into the immunohistochemical characteristics of breast cancer 117, 118, 119. For example, in a retrospective analysis of 171 patients, Groheux and colleagues demonstrated that SUVmax, SUVmean, and TLG were significantly associated with the three phenotype subgroups (triple‐negative, Her‐2‐positive, and ER‐positive/Her‐2‐negative breast cancer) 118. Koo et al. found that ^18^F‐FDG uptake was correlated with a high Ki‐67 index in triple‐negative breast cancer 117. PET based on ^18^F‐FES or ^89^Zr‐trastuzumab could even achieve dynamic monitoring of ER and Her‐2 status during concomitant endocrine and trastuzumab therapy. In a study by van Kruchten and colleagues, ^18^F‐FES PET was used to determine the dose needed for ER antagonists to completely abolish ER 120. Currin and coworkers demonstrated restoration of endocrine sensitivity after initial endocrine resistance with the help of ^18^F‐FES PET 121. In another study, Her‐2‐based PET was used to help explore heterogeneity during Her‐2 mapping of metastatic disease and to help select patients who may demonstrate a response to trastuzumab therapy 122. Several studies further demonstrated that PET successfully identified spatial metabolic heterogeneity in breast cancer 123. With the help of ^18^F‐FLT, intratumor proliferation heterogeneity was imaged using PET for assessing the response to neoadjuvant chemotherapy 124, 125.

Besides PET, QDs‐based imaging is also a promising molecular imaging technique (discussed earlier in the Pathologic Analysis section). QDs conjugated with various biomarkers could image different targets in breast cancer 77, 78, and they have shown great potential in molecular imaging. Tada and colleagues successfully applied anti‐Her‐2 antibody‐conjugated QDs to obtain images of Her‐2 overexpression in breast cancer xenografts 126. However, in vivo fluorescence imaging is affected by tissue depth. Some authors have tried to link QDs‐based imaging with other imaging techniques to solve this problem. For example, Ma and colleagues successfully achieved in vivo multimodality imaging using a multilayered, core/shell nanoprobe based on magnetic nanoparticles (MNPs) and QDs 127.

QDs‐based imaging is relatively immature for clinical application. However, QDs‐based imaging will have a future role in molecular imaging for breast cancer due to its optimal applicability in imaging acquisition and ability to quantify multiple biomarkers.

## Progress in Understanding of Heterogeneity

The technological advances reported in this paper have greatly increased our understanding of intertumor heterogeneity. Based on analysis of the copy number and gene‐expression profiling data from approximately 2000 breast cancer patients, Curtis et al. identified 10 novel molecular subgroups with distinct clinical outcomes 128. The 21‐gene assay based on gene‐expression profiling could provide additional prognostic information and predict benefit from adjuvant chemotherapy in ER‐positive patients 47. Another example is QDs‐IHC, with which we revealed intertumor heterogeneity pattern by quantitatively analyzing traditional biomarkers such as EGFR, Her‐2, and Ki‐67 81, 82. Furthermore, greater in‐depth knowledge of intratumor heterogeneity has resulted from these various new technologies. NGS offers the compelling advantage of providing an assessment of intratumor heterogeneity. A number of NGS studies demonstrated that breast tumors are composed of several subclones harboring different somatic mutations, copy number aberrations, and chromosomal rearrangements 19, 66. Further NGS analysis found that clonal competition and selective pressure from the microenvironment and therapy led to a state of dynamic change for these subclones 18, 22, 129. Using integrated NGS and digital RNA profiling, Balko and colleagues discovered that the genomic landscape of residual cancer after neoadjuvant chemotherapy was different from that of the pretreatment specimens, with increased enrichment in MCL1 amplification, PTEN deletions and/or mutations, JAK2 amplifications, and CDK6/CCND1–3 amplification 130. With the help of FISH, Janiszewska and colleagues found a dramatic increase in the relative frequency of PIK3CA‐mutant cells after neoadjuvant chemotherapy 27. Furthermore, NGS analyses of primary breast tumors and matched metastatic lesions have a distinct genomic landscape 131. Based on these findings, we can identify new therapeutic targets and develop a more detailed, targeted treatment plan. In actuality, ^18^F‐FES PET has been used to monitor endocrine resistance during endocrine therapy to help create more efficient treatment plans 121. However, overcoming intratumor heterogeneity will take considerably more research.

The microenvironment in which cancer cells exist is another important component of breast cancer and plays an essential role in cancer progression 15, and it can pose selective pressure on cancer cells by regulating cancer cell–signal transduction and gene expression. Developments in these techniques (mentioned above) also deepened our understanding of heterogeneity in a microenvironment. Conventional pathologic analysis and IHC have identified various stromal biomarkers such as COX‐2, MMP‐1, and Syndecan‐1 that are associated with breast cancer prognosis 132. Furthermore, with the help of QDs‐IHC, simultaneous imaging was possible of EGFR and collagen IV in triple‐negative breast cancer. EGFR and collagen IV could act as predictors of clinical outcomes 82. Genomic analysis was also a powerful tool. A 163‐gene prognosticator generated from stromal gene expression profiling was highly prognostic in Her‐2‐positive cases of breast cancer 133.

## Future Perspectives

Precision medicine has received greater attention in recent years, especially in the field of cancer treatment 134. As part of this new approach, effective detection of heterogeneity in breast cancer, both inter‐ and intratumor, has become imperative. In terms of genomic analysis, new analysis techniques and genomic data mining that will enable us to identify new targets for therapy are the primary future directions of breast cancer treatment. With regard to pathologic analysis, quantitative analysis of the heterogeneity of tumor(s) in breast cancer will be an important element in finding factors that are associated with prognosis and sensitivity (or resistance) to therapy. Molecular imaging shows great promise in the detection of heterogeneity in breast cancer; this imaging technique could combine morphological and molecular heterogeneity with functional heterogeneity, enabling more in‐depth understanding of intratumor heterogeneity. Collectively, development of these techniques has the potential to promote considerable progress in precision medicine with respect to detection of heterogeneity in breast cancer, and potential improvements in treatment.

## Conflict of Interest

None declared.
